# Sleep-Dependent Consolidation of Rewarded Behavior Is Diminished in Children with Attention Deficit Hyperactivity Disorder and a Comorbid Disorder of Social Behavior

**DOI:** 10.3389/fpsyg.2017.00167

**Published:** 2017-02-08

**Authors:** Christian D. Wiesner, Ina Molzow, Alexander Prehn-Kristensen, Lioba Baving

**Affiliations:** Department of Child and Adolescent Psychiatry and Psychotherapy, School of Medicine, Christian Albrecht UniversityKiel, Germany

**Keywords:** ADHD, REM-sleep, memory consolidation, reward-activation model, probabilistic learning

## Abstract

Children suffering from attention-deficit hyperactivity disorder (ADHD) often also display impaired learning and memory. Previous research has documented aberrant reward processing in ADHD as well as impaired sleep-dependent consolidation of declarative memory. We investigated whether sleep also fosters the consolidation of behavior learned by probabilistic reward and whether ADHD patients with a comorbid disorder of social behavior show deficits in this memory domain, too. A group of 17 ADHD patients with comorbid disorders of social behavior aged 8–12 years and healthy controls matched for age, IQ, and handedness took part in the experiment. During the encoding task, children worked on a probabilistic learning task acquiring behavioral preferences for stimuli rewarded most often. After a 12-hr retention interval of either sleep at night or wakefulness during the day, a reversal task was presented where the contingencies were reversed. Consolidation of rewarded behavior is indicated by greater resistance to reversal learning. We found that healthy children consolidate rewarded behavior better during a night of sleep than during a day awake and that the sleep-dependent consolidation of rewarded behavior by trend correlates with non-REM sleep but not with REM sleep. In contrast, children with ADHD and comorbid disorders of social behavior do not show sleep-dependent consolidation of rewarded behavior. Moreover, their consolidation of rewarded behavior does not correlate with sleep. The results indicate that dysfunctional sleep in children suffering from ADHD and disorders of social behavior might be a crucial factor in the consolidation of behavior learned by reward.

## Introduction

Attention deficit hyperactivity disorder (ADHD) is characterized by developmentally inappropriate levels of inattention, hyperactivity, and impulsivity (American Psychiatric Association [APA], 2013). With a prevalence of 5–7%, ADHD is the most often diagnosed psychiatric disorder of early childhood (Polanczyk et al., 2007; Willcutt, 2012; Thomas et al., 2015). However, a majority of children admitted for treatment in psychiatric institutions suffers from comorbid disorders, younger inpatients most often from conduct disorders (CD) and oppositional defiant disorders (ODD) (Yoshimasu et al., 2012). ADHD itself is a risk factor for school failure, and ADHD in combination with CD/ODD has an even worse prognosis (Kessler et al., 2014). Disturbed reward processing is a key neuropsychological feature in ADHD and in addition to attention deficits, may be a crucial factor in school failure (Luman et al., 2010; Tripp and Wickens, 2012; Silvetti et al., 2013; Plichta and Scheres, 2014; Tomasi and Volkow, 2014). Moreover, impaired reward processing as indicated by aberrant prefrontal cortex activation is a predictor for the persistence of ADHD symptoms into adulthood (Wetterling et al., 2015). Here, we focus on the role of sleep-dependent consolidation of rewarded behavior in children suffering from ADHD. First, we summarize recent studies on sleep-dependent consolidation in ADHD and then we highlight some studies on reward processing in ADHD.

It has been firmly established that sleep fosters the consolidation of declarative and procedural memory in adults (Diekelmann and Born, 2010; Rasch and Born, 2013) and declarative memory in children (Wilhelm et al., 2012). Several studies support the hypothesis that the prospect of reward can foster sleep-dependent consolidation of declarative memories in healthy adults (Tucker et al., 2011; van Dongen et al., 2012; Feld et al., 2014). Perogamvros and Schwartz (2012) suggest that reward-related memories become reactivated during REM-sleep which in turn strengthens their consolidation. Children suffering from ADHD show impairment in subjective and objective measures of sleep quality (Cortese et al., 2009), sleep microstructure (Ringli et al., 2013; Akinci et al., 2015), increased daytime sleepiness (Wiebe et al., 2013), and a higher rate of sleep disorders like restless legs or periodic limb movements (Kirov and Brand, 2014). The risk for behavioral sleep problems seems to be especially high in ADHD-patients with comorbid internalizing or externalizing disorders (Lycett et al., 2015). Moreover, sleep disorders can cause ADHD-like symptoms (Fischman et al., 2015), and behavioral sleep interventions can help to alleviate symptoms in patients with ADHD (Hiscock et al., 2015). For example, a study by Kershavarzi and colleagues highlights the role of sleep in social behavior (Keshavarzi et al., 2014). The authors showed that in ADHD patients a sleep hygiene training as compared to a control condition and to healthy controls not only improved sleep, mood, and psychological functioning but also social relationships and social acceptance. In our previous studies, we found that children with ADHD show reduced sleep-associated consolidation of declarative memory (Prehn-Kristensen et al., 2011) and that the deficit is especially pronounced when emotionally relevant material is learned (Prehn-Kristensen et al., 2013). On the other hand, increasing slow oscillations during slow-wave sleep by transcranial oscillating, direct-current stimulation can improve declarative memory consolidation in children with ADHD (Prehn-Kristensen et al., 2014).

While the focus of research on sleep-associated consolidation in children has been on declarative and procedural memory (Wilhelm et al., 2012), the research on learning and memory in ADHD has emphasized reward learning as a core deficit (Luman et al., 2010; Silvetti et al., 2013). Aberrant reward processing in ADHD is intimately linked to the dopamine hypofunction of the prefrontal cortex and striatum and can in part be normalized by dopaminergic medication like methylphenidate (MPH; Tripp and Wickens, 2012; Volkow et al., 2012; Tomasi and Volkow, 2014). Deficits include altered sensitivity to reward and/or punishment in probabilistic learning tasks (Luman et al., 2010; Maia and Frank, 2011). In Patients suffering from ADHD and comorbid CD/ODD, the deficits in reward processing are more pronounced (Groen et al., 2013) or, with regard to reward learning, only prevalent in comorbid patients (Luman et al., 2015). In ADHD patients the acquisition of stimulus-response mappings by probabilistic feedback (Luman et al., 2015) and their reversal (Wetterling et al., 2015) seem to be intact, but the mechanisms of learning are abnormal (Frank et al., 2007; Wetterling et al., 2015). Moreover, ADHD patients perform better when continuous or frequent feedback is provided but worse when infrequent probabilistic feedback is provided (Luman et al., 2010). Based on the paradigms of Frank et al. (2007) and Swainson et al. (2000), we developed a probabilistic learning and reversal task appropriate for children using increasing frequencies of valid feedback. With reference to Perogamvros and Schwartz (2012), we hypothesize that sleep fixates the stimulus-response mapping previously learned by reward and punishment by reactivating dopaminergic pathways during REM sleep and thereby makes it resistant to remapping during reversal learning.

Here, we focus on severely affected ADHD patients who were treated as in-patients in our clinic. All of the patients suffered from a comorbid disorder of social behavior (CD or ODD). It has been argued that this group poses a diagnostic entity separate from pure ADHD (Banaschewski et al., 2003). These patients also pose a great challenge for the therapeutic and pedagogic team because their learning difficulties most often extend to learning from feedback. Since children with ADHD are known to show deficits in reward learning, as well as alterations in the sleep-associated memory consolidation, and sleep is assumed to support the consolidation of reward-associated memory, we expect that children with ADHD + CD/ODD show diminished sleep-dependent consolidation of rewarded behavior. To investigate reward learning we used a probabilistic, two-alternative forced choice task and - after a retention interval of sleep or wakefulness - reversed the reward contingencies to measure consolidation of the previously learned contingencies. Healthy controls are expected to consolidate the previously learned behavior stronger during sleep than during wake and hence show more resistance to relearning after sleep as compared to wake whereas patients with ADHD + CD/ODD are supposed to consolidate less, especially during sleep (hypothesis 1). Moreover, we expect a correlation between the amount of REM sleep and the amount of sleep-dependent consolidation in the healthy controls but not in the patients (hypothesis 2).

## Materials and Methods

### Participants

A sample of 34 children (17 ADHD + CD/ODD, 17 Controls) took part in the study. Healthy controls were recruited via advertisements in local journals. Patients were referred to our study from the Clinic of Child and Adolescent Psychiatry and Psychotherapy of the University of Kiel. All parents of the participants gave written informed consent. All children gave informed assent and they were reimbursed with a voucher for their participation. The study protocol was approved by the ethics committee of the medical faculty of the University of Kiel and followed the ethical standards of the Helsinki declaration.

According to DSM-IV-TR criteria (American Psychiatric Association [APA], 2013), all patients suffered from attention deficit hyperactivity disorder (15 patients with combined type, 314.01; two patients with predominantly inattentive type, 314.0). Furthermore, three patients suffered from comorbid CD (312.81) and 14 from oppositional defiant disorder (313.81). Two of the three patients suffering from CD also fulfilled the diagnostic criteria for an oppositional defiant disorder. One patient also suffered from enuresis (307.6). Six patients fulfilled the diagnostic criteria of a combined disorder of reading and written expression (315.0 and 315.2, ICD-10 F81.0) and five more patients had only subclinical symptoms. No further comorbidities were diagnosed. In all, 13 patients took MPH but discontinued medication 48 h (approximately 12 half-lives) prior to the experimental sessions. None of the control children suffered from any psychiatric disorder. To secure the diagnoses in the patients and to preclude any psychiatric disorders in the controls, all children and their parents were interviewed using a German translation of the Revised Schedule for Affective Disorders and Schizophrenia for School-Age Children: Present and Lifetime Version (K-SADS-PL;) (Kaufman et al., 1997; Delmo et al., 2000). Furthermore, the Child Behavior Checklist (CBCL) (Achenbach, 1991) was filled out by the parents to assess any psychiatric symptoms of their children. The patients received significantly higher ratings on the internalizing problems scale (*t*_32_ = 3.05, *p* = 0.005, *d* = 1.05; descriptive statistics are reported in **Table 1**) and especially on the externalizing problems scale (*t*_32_ = 9.02, *p* < 0.001, *d* = 3.09).

**Table 1:** Demographic and clinical characteristics of children.

	ADHD (*n* = 17)	Control (*n* = 17)				
	Mean (SEM)	Mean (SEM)	*t*	*df*	*p*	*d*
Age	11.3 (0.4)	11.1 (0.2)	0.49	32	0.629	0.17
PDS	3.5 (0.2)	3.2 (0.1)	1.12	32	0.270	0.39
IQ	102.4 (3.1)	108.8 (2.7)	–1.54	32	0.134	–0.53
DCS	39.8 (1.6)	44.7 (1.6)	–2.13	32	0.041	–0.73
SSR	25.3 (1.1)	21.8 (0.9)	2.47	32	0.019	0.85
CSHQ	43.7 (1.2)	39.0 (0.9)	3.21	32	0.003	1.10
CBCL int.	62.1 (2.0)	53.1 (2.2)	3.05	32	0.005	1.05
CBCL ext.	69.7 (1.2)	48.9 (2.0)	9.02	32	<0.001	3.09
CBCL total	70.5 (1.2)	50.7 (2.0)	8.68	26.5^∗^	<0.001	2.98

*ADHD, attention deficit hyperactivity disorder; SEM, standard error of mean; PDS, Pubertal Development Scale; IQ, intelligence quotient as measured by the Culture Fair Test 2; DCS, visuospatial memory as measured with the Diagnosticum für Cerebralschädigung; SSR, Sleep Self-Report for children; CSHQ, Children’s Sleep Habit Questionnaire for parents; CBCL int., internalizing behavior scale of the Child Behavior Checklist; CBCL ext., externalizing behavior scale of the Child Behavior Checklist; CBCL total, total score of the Child Behavior Checklist; ^∗^ If a Levene test indicated inhomogeneous variances we used the Cochran and Cox adjustment for the standard error and corrected the degrees of freedom according to Satterthwaite.*

Since ADHD with comorbid disorders of social behavior is more often diagnosed in boys than in girls (Nussbaum, 2012), only boys were included in the sample. The age of patients and healthy controls ranged from 8 to 12 years and did not differ between the groups (*t*_32_ = 0.49, *p* = 0.629, *d* = 0.17; for descriptive statistics see **Table 1**). As assessed using the Edinburgh Handedness Inventory (Oldfield, 1971), 15 of the patients and 16 of the healthy controls were right-handed. All participants had normal or corrected-to-normal vision. Candidates were excluded from the study if they had: (1) below average intelligence with an IQ < 85, as measured by the Culture Fair Intelligence Test Revised Version (CFT-20-R) (Weiß, 2006), (2) significant memory impairment as measured by the Diagnosticum für Cerebralschädigung (DCS) with scores below the 16th percentile (Lamberti and Weidlich, 1999), (3) advanced puberty as measured by Pubertal Development Scale (PDS; total score >7) (Watzlawik, 2009), (4) any medical condition or impairment that would interfere with the ability to participate in the study as assessed by interview, or (5) any sleep disorders. We screened for sleep disturbances using the parent-reported Children’s Sleep Habits Questionnaire (CSHQ) (Owens et al., 2000; Schlarb, 2011) and the children’s Sleep Self-Report questionnaire (SSR) (Schwerdtle et al., 2010). Moreover, the polysomnograms of the adaptation nights were examined for symptoms of sleep disorders by a trained sleep lab technician. No abnormal sleep patterns or sleep disorders were detected. The demographic and clinical characteristics of the remaining 34 participants are reported in **Table 1**. Patients and healthy controls were comparable with respect to IQ (*t*_32_ = –1.54, *p* = 0.134, *d* = –0.53) and pubertal state (*t*_32_ = 1.12, *p* = 0.270, *d* = 0.39), but the patients showed lower memory performance in the DCS (*t*_32_ = –2.13, *p* = 0.041, *d* = –0.73) and worse sleep quality reported in the SSR (*t*_32_ = 2.47, *p* = 0.019, *d* = 0.846) or the CSHQ (*t*_32_ = 3.21, *p* = 0.003, *d* = 1.10).

### Probabilistic Learning and Reversal Task

**FIGURE 1:**
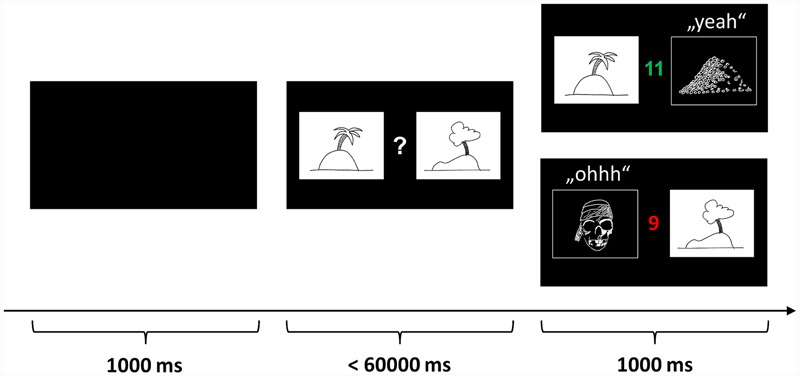
**Probabilistic learning and reversal task (“pirate game”).** In each trial, two pictures of islands are presented and the participant has to decide which island to explore. If the “correct” island is chosen, the picture of the island is replaced by a picture of a treasure, a sound of children cheering “yeah” is played, and the treasure counter is colored green and incremented by one (reward). If the “wrong” island is chosen, the island is replaced by a jolly roger, a disappointed voice uttering “ohhh” is played, and the treasure counter is colored red and decremented by one (punishment). The participants were instructed to learn by trial and error to approach the island on which a treasure is hidden more often and to avoid the island which is inhabited by pirates more often. The pictures above are merely symbolic. The actual pictures were color photos sampled from the internet.

In the so-called “pirate game” the children are asked to explore treasure islands (see **Figure 1**). Two equivalent versions of the pirate game with different stimulus sets were programmed in Presentation^®^ software (Version 14.9, Neurobehavioral Systems inc.) and the versions were approximately counterbalanced over conditions and order. In each trial, two pictures of islands are presented and the child has to decide which island to explore. Participants indicate their choice by pressing the corresponding mouse buttons. If the “correct” island is chosen, the picture of the island is replaced by a picture of a treasure, a sound of children cheering “yeah” is played, and the treasure counter is colored green and incremented by one (reward). If the “wrong” island is chosen, the island is replaced by a jolly roger, a disappointed voice uttering “ohhh” is played, and the treasure counter is colored red and decremented by one (punishment). During a block of 33 trials, the same islands are repeatedly shown on the left or right side of the monitor in pseudorandom, counterbalanced order. The participants were instructed to learn by trial and error to approach the island on which a treasure is hidden more often and to avoid the island which is inhabited by pirates more often. Probabilistic feedback was provided according to a reinforcement schedule with increasingly valid feedback: In the first third of the trials, the target island was correct with a frequency of 7/11 (≈63.6%) and wrong with a frequency of 4/11 (≈36.4%). In the second third the reward frequency was increased to 8/11 (≈72.7%) and the punishment frequency decreased to 3/11 (≈27.3%). Finally, in the last third, the reward frequency reached 9/11 (≈81.8%) and the punishment frequency 2/11 (≈18.2%). This schedule was chosen to allow the assessment of performance differences between the groups in the beginning of each block while ensuring that both groups encode the “correct” island equally well until the end of the block (compare results section).

During the encoding session before the retention interval, the children played three blocks of 33 trials (encoding). In each block, a unique set of island pictures was used. After the retention interval containing sleep or wake, the children played three blocks with entirely new picture sets (learning) to obtain a control measure for the influence of sleep vs. wake on learning performance. The crucial reversal learning block was identical to one of the encoding blocks using the same islands and the same reinforcement frequencies (reversal). However, the reinforcement schedule was reversed: now, formerly “correct” islands were “wrong” and vice versa. The rationale behind the reversal learning block was to test whether sleep helps to consolidate the stimulus-response mapping and therefore make it harder to reverse it during reversal learning. In other words, participants are expected to persist in preferring the formerly “correct” island as an indicator of the consolidation of rewarded behavior.

### Sleep Recording

All participants spent an adaptation night and a test night in the sleep laboratory separated by at least one night for recovery from potential sleep loss during the first night. The adaptation night’s purpose was to exclude severe sleep disorders and to help participants adapt to the conditions in the sleep laboratory. During both nights sleep was recorded by standard procedures using a digital electroencephalogram (EEG), electromyogram (EMG) and electrooculogram (EOG). To amplify and record the data, a SOMNOscreen PSG plus (SOMNOmedics, Randersacker, Germany) was used. The EEG was recorded at a sampling rate of 128 Hz with a band-pass filter of 0.2–35 Hz using multi-use Ag/AgCl-electrodes attached to the positions C3 and C4 according to the 10–20 system referenced to an electrode on the bridge of the nose and with a ground placed at Fpz. A diagonal EOG was recorded at a sampling rate of 128 Hz with a band-pass filter of 0.2–75 Hz using single-use Ag/AgCl-electrodes attached to the lower right and upper left canthi. A bipolar EMG was recorded at a sampling rate of 256 Hz with a band-pass filter of 0.2–128 Hz using single-use Ag/AgCl-electrodes attached to the chin. Only during the adaptation night did we additionally record an EMG from the anterior tibial muscles, a bipolar electrocardiogram, nasal air flow using a thermistor, and respiratory thorax excursions using a belt sensor. All sleep data were visually scored according to the criteria by Rechtschaffen and Kales (1968) by a certified rater unaware of the hypotheses. The following macro-sleep parameters were obtained: sleep stages 1 to 4 and REM sleep (in min), time in bed (in min), lights off, lights on, sleep-onset latency (time in min from lights off to first epoch of sleep stage 2), total sleep time (in min), sleep efficiency (ratio of total sleep time to time in bed in percent), number of awakenings, and duration of wakefulness after sleep onset (in min).

To control for effects of sleep on wakefulness and mood, the participants kept sleep logs, rated their wakefulness on a visual analog scale (ranging from 0 to 100) and their mood on the valence and arousal scales of the self-assessment manikin (SAM) (Bradley and Lang, 1994) in the morning and in the evening in the sleep as well as the wake condition. Furthermore, before every encoding or reversal session, the alertness test from the test battery of attentional performance for children (KiTAP) (Zimmermann et al., 2002) was administered.

### Procedure

The participants took part in two diagnostic sessions and two experimental conditions, each comprised of two sessions with an interposed delay of 12 h. During the first diagnostic session, the children and their parents were interviewed independently by trained psychologists, and tests and questionnaires were administered. The second diagnostic session was the adaptation night and took place at least two days before the experimental sleep condition. In the experimental wake condition the children arrived at the laboratory at 8:00 a.m., filled out the sleep log and rating scales, completed the alertness test, and played three blocks (i.e., pairs of islands) of the pirate game (encoding). The participants were instructed not to sleep during the day and attend to their usual daily routines. Twelve hours later (8:00 p.m.) the children came back to the laboratory, filled out log and rating scales again, performed the alertness test, and played three new blocks of the pirate game (learning) as well as on one block with reversed contingencies (reversal).

In the sleep condition, the children arrived at the laboratory at 8 p.m. on the day of the experimental night and worked on the logs, scales, test and pirate game as described above. The electrodes for the PSG were attached around 9 p.m., lights off was at 9:30 p.m. and lights on at 7:00 a.m. Again, the second part of the testing took place 12 h after encoding. The sleep and the wake conditions were at least two weeks apart and the conditions and parallelized stimulus sets were approximately counterbalanced over groups.

### Data Processing and Statistical Analysis

First, we describe the preprocessing of the behavioral data from the probabilistic learning and reversal task. Second, we delineate the inference statistics and how we dealt with the control variables.

**FIGURE 2:**
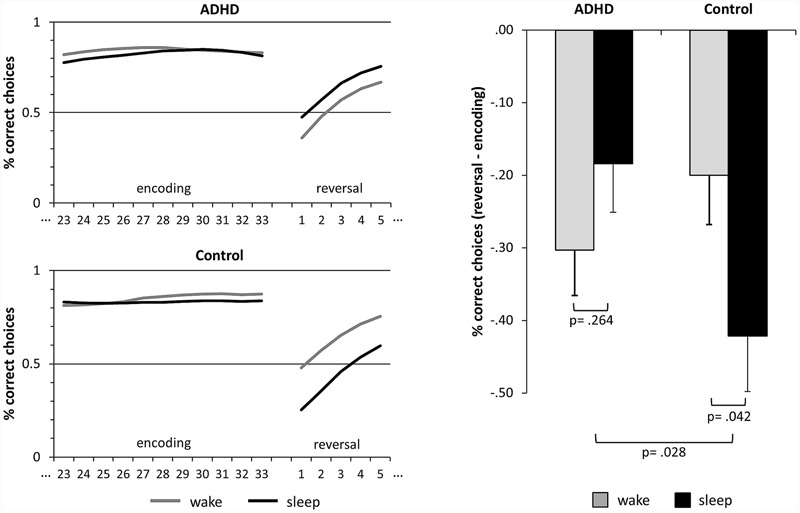
**Sleep-dependent consolidation of rewarded behavior.** The line graphics on the left side depict the relative frequencies of correct choices of the target islands. Encoding refers to the last thirds of the learning blocks prior to the retention intervals. After the retention intervals, the reinforcement schedule is reversed and the formerly “correct” islands become “wrong” and vice versa. Reversal refers to the first five trials of the reversal block after the retention interval. The bar graph on the right side depicts the difference of reversal and encoding. Larger negative values indicate a consolidation of the learned preferences, i.e., consolidation of rewarded behavior. The bars represent means ± standard error of means. ADHD, attention deficit hyperactivity disorder.

In the probabilistic learning paradigm, we counted the choices of the “correct” target stimulus which was followed by reward more often. In a few trials, some participants clicked the mouse multiple times so that responses were carried over to the next trial. These responses, indicated by reaction times shorter than 100 ms in the following trial, were deleted and interpolated with a random choice. The patients showed significantly more multiple button presses than healthy controls (*t*_16.8_ = 2.19, *p* = 0.043, *d* = 0.75). However, multiple reactions were very scarce, amounting to only 0.65% ± 0.27% (MEAN ± SEM) in the patients and 0.06% ± 0.04% in the healthy controls. The resulting learning curves were then filtered with a two-way moving average with a window size of five to reduce random noise in the choice data (Smith, 1997). To measure encoding success, we calculated the relative frequency of correct choices during the last thirds of the three encoding blocks (see **Figure 2**). To obtain a measure for the consolidation of the stimulus-response mappings, we calculated the difference of the relative frequency of correct responses during the first five trials of the reversal learning block and the encoding success (reversal – encoding; see **Figure 2**). To control for influences of time of day on learning performance *per se*, we calculated the correct responses during the first five trials of the three new learning blocks after the retention interval.

To control whether the participants learned to prefer the island associated with reward more often during encoding, we computed *t*-tests comparing the performance during the last thirds of the three encoding blocks against guessing frequency (0.5). To evaluate whether sleep effected the consolidation of learned behavior differently in the ADHD + CD/ODD patients compared to healthy controls, we calculated an ANOVA using SLEEP (sleep vs. wake) and LEARNING (encoding vs. reversal) as within-subject factors and GROUP (ADHD vs. control) as a between-subject factor. Significant effects were resolved using Bonferroni-adjusted *post hoc* contrasts. Also, the difference between the performance during encoding and reversal in the sleep condition as a measure of sleep-dependent memory retention was correlated with the amounts of REM sleep and non-REM sleep. To control for differences in learning performance, we computed an ANOVA of the control measure learning using SLEEP as within and GROUP as between factors. To test whether the manipulation had any effect on subjective wakefulness, valence, arousal or objective alertness and learning performance, we computed ANOVAs with TIME (morning vs. evening) as a within-subject and GROUP (ADHD vs. control) as a between-subject factor with *post hoc* contrasts. In the case of a significant main effect of GROUP or a significant interaction of TIME and GROUP, the main analysis of the data from the probabilistic learning and reversal task was repeated using the respective control variables as covariates. To compare the groups regarding sleep parameters and questionnaire data, *t*-tests were used. Descriptives are reported as a mean ± standard error of the mean (SEM). For all *t*-tests, Cohen’s *d* was computed as a measure of effect size. Pearson’s correlation coefficients and partial η^2^ from the analyses of variance were converted to *d* values according to Cohen (1988) and Rosenthal (1994).

## Results

### Sleep

Children in both groups slept roughly 8 h during the experimental night (ADHD: 475.6 ± 9.0, Control: 482.7 ± 8.9, *t*_32_ = –0.56, *p* = 0.578, *d* = –0.19). The amount of sleep in the sleep stages 1 to 4 and REM sleep did not differ between groups (all *p* > 0.213, also see **Table 2**). By trend, the amount of slow-wave sleep (S3 + S4) was slightly higher in the control group (117.0 ± 5.5) than in the patients (105.3 ± 3.8, *t*_32_ = –1.75, *p* = 0.091, *d* = –0.60). All sleep parameters were within normal range (Scholle et al., 2011).

**Table 2:** Sleep parameters across groups.

	ADHD	Control				
	Mean (SEM)	Mean (SEM)	*t*	*df*	*p*	*d*
Time in bed (min)	530.0 (7.1)	543.1 (6.4)	–1.38	32	0.179	-0.47
Total sleep time (min)	475.6 (9.0)	482.7 (8.9)	–0.56	32	0.578	-0.19
Sleep efficiency (%)	89.7 (1.2)	88.9 (0.9)	0.52	32	0.609	0.18
Sleep onset lat. (min)	19.5 (3.8)	25.2 (4.5)	–0.96	32	0.345	-0.33
REM sleep lat. (min)	104.6 (11.6)	107.8 (8.1)	–0.22	32	0.825	-0.08
Awake (min)	48.1 (6.4)	53.6 (4.6)	–0.70	32	0.490	-0.24
S1 (min)	39.3 (3.9)	45.7 (3.2)	–1.27	32	0.213	-0.44
S2 (min)	233.6 (6.3)	220.0 (11.4)	1.05	32	0.300	0.36
S3 (min)	41.7 (2.8)	40.4 (1.8)	0.39	32	0.702	0.13
S4 (min)	69.2 (4.5)	76.6 (4.6)	–1.16	32	0.256	-0.40
Non-REM sleep (min)	344.5 (7.3)	338.7 (9.3)	0.49	32	0.627	0.17
REM sleep (min)	91.7 (4.8)	96.5 (4.4)	–0.73	32	0.468	-0.25

*ADHD, attention deficit hyperactivity disorder; SEM, standard error of the mean; S1–S4 and REM sleep rated according to the criteria of Rechtschaffen and Kales (1968).*

### Probabilistic Learning and Reversal

All participants learned to prefer the island associated with reward more often during encoding. The relative frequencies of correct choices during the last thirds of the encoding blocks were significantly higher than the guessing frequency 0.5 in healthy controls (prior to wake: *t*_16_ = 10.15, *p* < 0.001, *d* = 2.46 /prior to sleep: *t*_16_ = 8.59, *p* < 0.001, *d* = 2.08) as well as in patients (prior to wake: *t*_16_ = 10.54, *p* < 0.001, *d* = 2.56/prior to sleep: *t*_16_ = 9.56, *p* < 0.001, *d* = 2.32, also see **Table 3** and **Figure 2**). Please note that on a descriptive level all values of individual participants were greater than the guessing frequency 0.5. This confirms that all participants encoded the rewarded behavior, namely the preference for the correct island.

**Table 3:** Encoding and reversal performance.

	ADHD			Control		
	Wake Mean (SEM)	Sleep Mean (SEM)	Difference Mean (SEM)	Wake Mean (SEM)	Sleep Mean (SEM)	Difference Mean (SEM)
Encoding	0.846 (0.034)	0.818 (0.036)	0.027 (0.028)	0.850 (0.034)	0.829 (0.036)	0.021 (0.036)
Reversal	0.543 (0.053)	0.634 (0.067)	-0.092 (0.090)	0.650 (0.053)	0.408 (0.067)	0.242 (0.114)
Difference	0.303 (0.065)^∗∗∗^	0.184 (0.072)+	0.119 (0.095)	0.200 (0.065)^∗^	0.421 (0.072)^∗∗∗^	-0.221 (0.114)^∗^
Effect size *d*	1.12	0.62	0.28	0.74	1.42	-0.51

*Relative frequencies of correct choices of the target islands are reported. Encoding refers to the learning blocks prior to the retention intervals. After the retention intervals, during reversal learning the reinforcement schedule is reversed and the formerly “correct” islands become “wrong” and vice versa. ADHD, attention deficit hyperactivity disorder; SEM, standard error of mean; ^+^*p* = 0.055; ^∗^*p* < 0.05; ^∗∗∗^*p* < 0.001.*

The performance was significantly higher at the end of the encoding blocks as compared to the beginning of the reversal block (main effect of LEARNING, *F*_1,32_ = 76.90, *p* < 0.001, *d* = 3.10; also see **Table 3** and **Figure 2**). This illustrates that the previously learned preferences were retained in memory and had to be relearned during reversal. There were no main effects of GROUP (*F*_1,32_ = 0.68, *p* = 0.417, *d* = 0.29) or SLEEP (*F*_1,32_ = 1.61, *p* = 0.214, *d* = 0.49) on the performance as well as no interaction effects of LEARNING and SLEEP (*F*_1,32_ = 0.48, *p* = 0.495, *d* = 0.24) or LEARNING and GROUP (*F*_1,32_ = 1.12, *p* = 0.298, *d* = 0.37). However, there was a significant interaction effect of SLEEP and GROUP (*F*_1,32_ = 4.39, *p* = 0.044, *d* = 0.74) which was qualified by a significant three-way interaction of SLEEP, LEARNING and GROUP (*F*_1,32_ = 5.28, *p* = 0.028, *d* = 0.81). Comparing the performance of encoding and reversal by *post hoc* contrasts, we found that performance of the control participants dropped under the wake (*p* = 0.027, *d* = 0.74) as well as under the sleep condition (*p* < 0.001, *d* = 1.42). In patients performance significantly dropped in the wake (*p* < 0.001, *d* = 1.12) and, by trend, in the sleep condition (*p* = 0.094, *d* = 0.62). Again, this confirms that in general contingencies were retained in memory in both groups under both conditions. However, the drop of performance did not differ between sleep and wake in the patients (*p* = 0.264, *d* = 0.28) but was stronger during sleep than during wake in the control participants (*p* = 0.042, *d* = –0.51), and this double difference was significant (*p* = 0.028, *d* = 0.79, see **Figure 2**). Hence, only the control participants showed sleep-dependent consolidation of rewarded behavior (hypothesis 1).

Note that learning performance *per se* did not differ between conditions or groups. The ANOVA of the additional control measure learning revealed no main effect of SLEEP (*F*_1;32_ = 0.01, *p* = 0.930, *d* = 0.03), no main effect of GROUP (*F*_1,32_ = 1.47, *p* = 0.234, *d* = 0.43), and no interaction of SLEEP and GROUP (*F*_1,32_ = 1.41, *p* = 0.243, *d* = 0.42). This also illustrates that time of day did not influence learning performance as this would have produced a main effect of condition (SLEEP) in this control measure. Furthermore, our data preprocessing did not bias the results: The interaction effect reported above would remain significant (*p* = 0.026, *d* = 0.86) if the two outlier-patients with the highest rate of multiple reactions were excluded from the analysis. Filtering slightly improved the significance of the interaction effect (*p* = 0.044 to *p* = 0.028/*d* = 0.74 to *d* = 0.81) but did not change the results.

It was assumed that REM sleep fosters the consolidation of rewarded behavior in healthy controls (Perogamvros and Schwartz, 2012). Therefore, REM sleep was expected to correlate with the magnitude of the drop in performance during the night, whereas non-REM sleep should not (hypothesis 2). In ADHD there were only non-significant correlations of the performance drop with REM sleep (*r* = 0.322, *n* = 17, *p* = 0.207, *d* = 0.68) and non-REM sleep (*r* = 0.138, *n* = 17, *p* = 0.597, *d* = 0.28), and the difference between these correlations was not significant either (*z* = 0.640, *p* = 0.261). In contrast, the control participants showed a significantly higher (*z* = –1.819, *p* = 0.034) correlation of performance drop with non-REM sleep (*r* = 0.441, *n* = 17, *p* = 0.076, *d* = 0.98) than with REM sleep (*r* = –0.233, *n* = 17, *p* = 0.368, *d* = –0.48). Finally, we analyzed REM latency and REM density but did not find any significant correlations with the consolidation of rewarded behavior in healthy controls (all *p* > 0.276, all *d* < | 0.58|) or in patients (all *p* > 0.215, all *d* < | 0.61|).

### Control Variables

To exclude the possible interpretation that the diminished sleep-dependent consolidation of rewarded behavior is caused by overall psychopathology we repeated the analysis reported above using LEARNING and SLEEP as within-subject factors, GROUP as a between-subject factor and the overall score of the CBCL a covariate. However, the three-way interaction reported remains significant (*p* = 0.015, *d* = 0.94). The same is true when we use the score of the nonverbal memory test DCS as a covariate (*p* = 0.038, *d* = 0.78).

Furthermore, we controlled for effects of TIME of day on alertness and subjective valence, arousal and wakefulness. The TIME of day (*F*_1,32_ = 6.90, *p* = 0.013, *d* = 0.93) and GROUP (*F*_1,32_ = 6.12, *p* = 0.019, *d* = 0.87) had an impact on the reaction times in the alertness test and there was a trend toward an interaction (*F*_1,32_ = 3.39, *p* = 0.075, *d* = 0.65). *Post hoc* contrasts revealed that patients were slower than controls in the mornings (*p* = 0.046, *d* = –0.71) and in the evenings (*p* = 0.014, *d* = –0.90; descriptive data not shown). Furthermore, patients slowed down during the day (*p* = 0.003, *d* = –0.62) as opposed to the controls (*p* = 0.582, *d* = –0.20). Therefore, we used the performance in the alertness test in the morning and evening as covariates in an ANCOVA of the performance in the probabilistic learning and reversal task. However, the interaction effect reported above stays stable (*F*_1,30_ = 5.87, *p* = 0.022, *d* = 0.89).

Neither TIME of day nor GROUP had any influence on the valence (all *p* > 0.159, all *d* < 0.51) or arousal ratings (all *p* > 0.139, all *d* < 0.54). However, the analysis of the subjective wakefulness revealed a significant interaction of TIME of day and GROUP (*F*_1,32_ = 9.60, *p* = 0.004, *d* = 1.10) but no main effects (all *p* > 0.270, *d* < 0.40). *Post hoc* contrasts showed that the groups’ wakefulness did not differ in the mornings (*p* = 0.123, *d* = –0.54) but in the evenings (*p* = 0.047, *d* = 0.71) and that the patients’ wakefulness declined during the day (*p* = 0.005, *d* = –0.71) as opposed the controls’ wakefulness (*p* = 0.172, *d* = 0.35). Again, we used the subjective wakefulness in the mornings and evenings as covariates in an ANCOVA of the performance in the probabilistic learning and reversal task. The interaction effect reported above was not significant any longer (*F*_1,30_ = 2.50, *p* = 0.125, *d* = 0.58) but on a descriptive level, the adjusted means pointed in the same direction as reported above.

## Discussion

It is important to shed some more light on the learning difficulties of children suffering from ADHD, especially those with the worst prognosis affected by comorbid disorders of social behavior. In our study, we focused on the consolidation of behavior learned by reward and the role of sleep in the consolidation process. We found that typically developing control children consolidate rewarded behavior better during a night of sleep than during a day awake and that the sleep-dependent consolidation of rewarded behavior by trend correlated with non-REM sleep but not with REM sleep. In contrast, children with ADHD and comorbid disorders of social behavior do not show sleep-dependent consolidation of rewarded behavior (hypothesis 1). Moreover, their retention of rewarded behavior over sleep did not correlate with sleep, especially not with REM sleep (hypothesis 2).

Our results extend our previous studies, in which we showed that sleep promotes the consolidation of declarative memory in healthy children but not in children with ADHD (Prehn-Kristensen et al., 2011) and that this deficit is especially pronounced for emotional declarative memory (Prehn-Kristensen et al., 2013). The current study extends this conclusion to the domain of reward learning: Sleep seems to foster the consolidation of rewarded behavior in healthy children but not in children suffering from ADHD + CD/ODD (hypothesis 1). We did not find any main effects of group on learning performance or resistance to reversal learning. This parallels another study using instrumental learning with probabilistic feedback where patients with ADHD showed normal learning curves (Luman et al., 2015). Furthermore, a study using probabilistic reversal learning also did not find differences between ADHD patients and controls during reversal, but altered activation of the frontostriatal reward network in ADHD patients predicted whether the symptoms of ADHD persisted (Wetterling et al., 2015). This highlights the necessity of looking at the functionality of brain activity during the consolidation of rewarded behavior in ADHD. In our study, we made a first step by showing that sleep-dependent consolidation of rewarded behavior is diminished in ADHD.

It could be argued that typically patients with ADHD show less motivation and/or a greater tendency to perseverate than the healthy controls and that this could produce slower relearning during reversal. However, there are several controls implemented in the design of our study: A lack of motivation would have caused a main effect of the factor GROUP regarding the encoding performance. However, the groups did not differ regarding the performance during encoding nor during the additional encoding control task. Moreover, the main result of our study is a three-way interaction of the between-subject factor GROUP and the within-subject factors SLEEP (wake vs. sleep) and LEARNING (encoding vs. reversal). We calculated several ANOVAs and ANCOVAs to demonstrate the robustness of this result. If any distinctive feature of the patient group, like a lack of motivation or a tendency to perseveration, would have influenced the performance in our paradigm it would have produced a main effect of GROUP or an interaction of GROUP and LEARNING but not a three-way interaction of GROUP, LEARNING, and SLEEP. Therefore we assume that dysfunctional sleep caused the three-way interaction, supporting our claim that sleep fosters the consolidation of rewarded behavior in healthy children but not in children suffering from ADHD + CD/ODD.

According to the reward activation model by Perogamvros and Schwartz (2012), the ventral tegmental area is activated predominantly during REM sleep and replays neural burst firing patterns associated with reward processing, thereby fostering memory consolidation. In essence, we would have expected a strong correlation between the amount of REM sleep and the consolidation of rewarded behavior (hypothesis 2). Contrary to expectations, the consolidation of rewarded behavior showed a stronger correlation with non-REM sleep than with REM sleep only in healthy children. Furthermore, the consolidation of rewarded behavior did not correlate with REM latency or REM density. Therefore, we cannot confirm the reward activation model. Instead, the correlation of the consolidation of rewarded behavior with non-REM sleep may be attributed to explicit aspects of the task. Patients suffering from anterograde episodic amnesia can still implicitly learn using probabilistic reward, but it has also been shown that explicit knowledge of the task structure facilitates learning (Speekenbrink et al., 2008). Furthermore, it has been firmly established that non-REM sleep, especially slow wave sleep, fosters the consolidation of declarative memory (Diekelmann and Born, 2010). Therefore, explicit aspects of the task might have been consolidated during non-REM sleep in healthy children.

Although the macroscopic sleep parameters investigated in this study did not differ between ADHD + CD/ODD patients and controls, the functional role of sleep in the consolidation of rewarded behavior seems to be impaired in the patients. This is in accord with our previous research which did not show differences in macroscopic sleep parameters between ADHD patients and controls either (Prehn-Kristensen et al., 2013). However, in a study utilizing transcranial oscillatory direct current stimulation during sleep, we were able to experimentally increase slow oscillation power during S4 in children with ADHD which was accompanied by a normalization of sleep-dependent consolidation of declarative memory (Prehn-Kristensen et al., 2014). Since in the present study we found a trend toward a correlation between sleep-dependent consolidation of rewarded behavior and non-REM sleep in healthy children, it seems likely that parameters like slow oscillations or sleep spindles might be involved. This would match a recent study, in which fast sleep spindles and delta power during non-REM sleep were shown to help in the development of procedural skills (Fogel et al., 2015). In future studies the amount of REM sleep and slow-wave sleep in children suffering from ADHD + CD/ODD should be manipulated – e.g., using the split-night paradigm (Yaroush et al., 1971) – to further assess the role of REM sleep and non-REM sleep as well as their accompanying waveforms on the consolidation of rewarded behavior.

A limitation of our study results from the comorbidity. It has been argued that patients suffering from ADHD and a disorder of social behavior pose a diagnostic entity separate from patients with ADHD but without a disorder of social behavior (Banaschewski et al., 2003). This was also reflected in the ICD-10 diagnosis hyperkinetic CD (F90.1). Therefore our results cannot be extended to all presentations of ADHD as defined by the DSMIV-TR or the DSM-V. However, previous studies also found diminished sleep-dependent consolidation of declarative memories in ADHD patients without CD/ODD (Wilhelm et al., 2012). In the present study, we focus on severely affected ADHD patients with CD/ODD because they pose a great challenge for behavior therapists due to their difficulties to learn from feedback. Future studies should investigate whether sleep-dependent consolidation of rewarded behavior is also diminished in lighter forms of pure ADHD.

Further limitations of the study could arise from circadian influences on learning performance, alertness, mood (valence, arousal), and wakefulness. However, we did not find any effects of time of day or group on learning performance or mood. As expected, ADHD + CD/ODD patients showed slower reaction times in the alertness test and slowed down even more during the day. However, the results concerning the consolidation of rewarded behavior did not change when we entered the alertness scores into an ANCOVA. Furthermore, on the descriptive level the ADHD + CD/ODD patients showed better memory retention during the day than during the night, a fact which cannot be explained by decreasing alertness during the day. ADHD + CD/ODD patients’ subjective wakefulness declined more rapidly than that of the controls. The ANCOVA showed that subjective wakefulness explained some variance in the consolidation of rewarded behavior, but the direction of the effect remained constant. Here again, the decline in wakefulness during the day in ADHD + CD/ODD patients cannot explain why they showed better memory retention during the day than during the night. Therefore, it seems unlikely that our results are due to circadian effects on learning performance, alertness, mood, or wakefulness. However, in future studies, hormonal changes should be taken into account because ADHD patients display a delayed melatonin dim-light onset and a flatter slope of the cortisol profile, both of which are probably related to sleep and memory (Imeraj et al., 2012; Bijlenga et al., 2013).

A limitation of our study is the lack of significant single correlations of sleep-dependent consolidation of rewarded behavior with sleep parameters. Only in healthy children did the correlation of consolidation with non-REM sleep approach significance. Our conclusion that non-REM sleep is more important for sleep-dependent consolidation of rewarded behavior rests mainly on the significant differences in correlations. On the other hand, the lack of significant correlations of consolidation with REM sleep does not confirm the reward activation model either. In any case, experimental manipulations of sleep stages using the split-night design or selective sleep deprivation could substantially add to the picture (Wiesner et al., 2015).

To our knowledge, ours is the first study investigating the sleep-dependent consolidation of rewarded behavior in ADHD + CD/ODD. Severely affected patients pose a great challenge for therapeutic and pedagogical interventions. Behavioral therapy using reward is the main approach in these patients. Therefore the consolidation of behavior learned by reward is a topic of high clinical relevance. In summary, our results indicate that healthy children consolidate rewarded behavior better during a night of sleep than during a day awake. Furthermore, sleep-dependent consolidation of rewarded behavior in healthy children correlates by trend with non-REM sleep but not with REM sleep. In contrast, sleep-dependent consolidation of rewarded behavior is diminished in children with ADHD and does not correlate with sleep. This could help to explain why children suffering from ADHD often display impaired learning and memory and are at risk of school failure. Moreover, impaired consolidation of behavior learned by feedback might be a reason why children with ADHD do not adopt newly learned social skills in everyday life as seen in healthy children. Therefore, we recommend taking into account poor sleep quality when treating children with ADHD and a comorbid disorder of social behavior. As Keshavarzi et al. (2014) pointed out, a sleep hygiene training might help to improve both sleep as well as social behavior in children with ADHD.

## Ethics Statement

This study was carried out in accordance with the recommendations of Declaration of Helsinki. All parents of the participants gave written informed consent. All children gave written, informed assent. The study protocol was approved by the ethics committee of the medical faculty of the University of Kiel and followed the ethical standards of the Helsinki declaration.

## Author Contributions

CW, IM, AP-K, LB designed the study. CW programmed the software. IM and AP-K collected the data. CW, IM, AP-K, LB analyzed and interpreted the data. CW wrote the manuscript. IM, AP-K, LB revised the manuscript. CW, IM, AP-K, LB approved the manuscript. CW, IM, AP-K, LB agreed to be accountable for all aspects of the work.

## Conflict of Interest Statement

The authors declare that the research was conducted in the absence of any commercial or financial relationships that could be construed as a potential conflict of interest.

## References

[B1] Achenbach T. M. (1991). *Manual for the Child Behavior Checklist/4-18 and 1991 Profile*. Burlington,VT: University of Vermont.

[B2] Akinci G. Oztura I. Hiz S. Akdogan O. Karaarslan D. Ozek H. (2015). Sleep structure in children with attention-deficit/hyperactivity disorder. *J. Child Neurol.* 30 1520–1525. 10.1177/0883073815573318 25713005

[B3] American Psychiatric Association [APA] (2013). *Diagnostic and Statistical Manual of Mental Disorders DSM-V.* Arlington, VA: American Psychiatric Publishing.

[B4] Banaschewski T. Brandeis D. Heinrich H. Albrecht B. Brunner E. Rothenberger A. (2003). Association of ADHD and conduct disorder–brain electrical evidence for the existence of a distinct subtype. *J. Child Psychol. Psychiatry* 44 356–376. 10.1111/1469-7610.00127 12635966

[B5] Bijlenga D. Van Someren E. J. Gruber R. Bron T. I. I Kruithof F. Spanbroek E. C. (2013). Body temperature, activity and melatonin profiles in adults with attention-deficit/hyperactivity disorder and delayed sleep: a case-control study. *J. Sleep Res.* 22 607–616. 10.1111/jsr.12075 23952346

[B6] Bradley M. M. Lang P. J. (1994). Measuring emotion: the self-assessment Manikin and the semantic differential. *J. Behav. Ther. Exp. Psychiatry* 25 49–59. 10.1016/0005-7916(94)90063-9 7962581

[B7] Cohen J. (1988). *Statistical Power Analysis for the Behavioral Sciences.* Hillsdale, NJ: Erlbaum.

[B8] Cortese S. Faraone S. V. Konofal E. Lecendreux M. (2009). Sleep in children with attention-deficit/hyperactivity disorder: meta-analysis of subjective and objective studies. *J. Am. Acad. Child Adolesc. Psychiatry* 48 894–908. 10.1097/CHI.0b013e3181ac09c9 19625983

[B9] Delmo C. Weiffenbach O. Gabriel M. Bölte S. Marchio E. Poustka F. (2000). *Fragebogen für Affektive Störungen und Schizophrenie für Kinder im Schulalter (6–18 Jahre)*. Frankfurt: Klinik für Psychiatrie und Psychotherapie des Kindes- und Jugendalters.

[B10] Diekelmann S. Born J. (2010). The memory function of sleep. *Nat. Rev. Neurosci.* 11 114–126. 10.1038/nrn2762 20046194

[B11] Feld G. B. Besedovsky L. Kaida K. Munte T. F. Born J. (2014). Dopamine D2-like receptor activation wipes out preferential consolidation of high over low reward memories during human sleep. *J*. *Cogn. Neurosci.* 26 2310–2320. 10.1162/jocn_a_00629 24669794

[B12] Fischman S. Kuﬄer D. P. Bloch C. (2015). Disordered sleep as a cause of attention deficit/hyperactivity disorder: recognition and management. *Clin. Pediatr. (Phila)* 54 713–722. 10.1177/0009922814548673 25187274

[B13] Fogel S. M. Ray L. B. Binnie L. Owen A. M. (2015). How to become an expert: a new perspective on the role of sleep in the mastery of procedural skills. *Neurobiol. Learn. Mem.* 125 236–248. 10.1016/j.nlm.2015.10.004 26477835

[B14] Frank M. J. Santamaria A. O’Reilly R. C. Willcutt E. (2007). Testing computational models of dopamine and noradrenaline dysfunction in attention deficit/hyperactivity disorder. *Neuropsychopharmacology* 32 1583–1599. 10.1038/sj.npp.1301278 17164816

[B15] Groen Y. Gaastra G. F. Lewis-Evans B. Tucha O. (2013). Risky behavior in gambling tasks in individuals with ADHD–a systematic literature review. *PLoS ONE* 8:e74909 10.1371/journal.pone.0074909 PMC3772864 24058638

[B16] Hiscock H. Sciberras E. Mensah F. Gerner B. Efron D. Khano S. (2015). Impact of a behavioural sleep intervention on symptoms and sleep in children with attention deficit hyperactivity disorder, and parental mental health: randomised controlled trial. *BMC. J.* 350:h68 10.1136/bmj.h68 PMC4299655 25646809

[B17] Imeraj L. Antrop I. Roeyers H. Swanson J. Deschepper E. Bal S. (2012). Time-of-day effects in arousal: disrupted diurnal cortisol profiles in children with ADHD. *J. Child Psychol. Psychiatry* 53 782–789. 10.1111/j.1469-7610.2012.02526.x 22324289

[B18] Kaufman J. Birmaher B. Brent D. Rao U. Flynn C. Moreci P. (1997). Schedule for affective disorders and schizophrenia for school-age children – Present and lifetime version (K-SADS-PL): initial reliability and validity data. *J. Am. Acad. Child Adolesc. Psychiatry* 36 980–988. 10.1016/S0890-8567(09)66571-0 9204677

[B19] Keshavarzi Z. Bajoghli H. Mohamadi M. R. Salmanian M. Kirov R. Gerber M. (2014). In a randomized case-control trial with 10-years olds suffering from attention deficit/hyperactivity disorder (ADHD) sleep and psychological functioning improved during a 12-week sleep-training program. *World J. Biol. Psychiatry* 15 609–619. 10.3109/15622975.2014.922698 24957753

[B20] Kessler R. C. Adler L. A. Berglund P. Green J. G. McLaughlin K. A. Fayyad J. (2014). The effects of temporally secondary co-morbid mental disorders on the associations of DSM-IV ADHD with adverse outcomes in the US National Comorbidity Survey Replication Adolescent Supplement (NCS-A). *Psychol. Med.* 44 1779–1792. 10.1017/S0033291713002419 24103255 PMC4124915

[B21] Kirov R. Brand S. (2014). Sleep problems and their effect in ADHD. *Expert Rev. Neurother.* 14 287–299. 10.1586/14737175.2014.885382 24491141

[B22] Lamberti G. Weidlich S. (1999). *DCS - A Visual Learning and Memory Test for Neuropsychological Assessment* 3rd Edn. Goettingen: Hogrefe.

[B23] Luman M. Goos V. Oosterlaan J. (2015). Instrumental learning in ADHD in a context of reward: intact learning curves and performance improvement with methylphenidate. *J. Abnorm. Child Psychol.* 43 681–691. 10.1007/s10802-014-9934-1 25212229

[B24] Luman M. Tripp G. Scheres A. (2010). Identifying the neurobiology of altered reinforcement sensitivity in ADHD: a review and research agenda. *Neurosci. Biobehav. Rev.* 34 744–754. 10.1016/j.neubiorev.2009.11.021 19944715

[B25] Lycett K. Sciberras E. Mensah F. K. Hiscock H. (2015). Behavioral sleep problems and internalizing and externalizing comorbidities in children with attention-deficit/hyperactivity disorder. *Eur. Child Adolesc. Psychiatry* 24 31–40. 10.1007/s00787-014-0530-2 24633694

[B26] Maia T. V. Frank M. J. (2011). From reinforcement learning models to psychiatric and neurological disorders. *Nat. Neurosci.* 14 154–162. 10.1038/nn.2723 21270784 PMC4408000

[B27] Nussbaum N. L. (2012). ADHD and female specific concerns: a review of the literature and clinical implications. *J. Atten. Disord.* 16 87–100. 10.1177/1087054711416909 21976033

[B28] Oldfield R. C. (1971). The assessment and analysis of handedness: the Edinburgh inventory. *Neuropsychologia* 9 97–113. 10.1016/0028-3932(71)90067-4 5146491

[B29] Owens J. A. Spirito A. McGuinn M. (2000). The Children’s Sleep Habits Questionnaire (CSHQ): psychometric properties of a survey instrument for school-aged children. *Sleep* 23 1043–1051. 11145319

[B30] Perogamvros L. Schwartz S. (2012). The roles of the reward system in sleep and dreaming. *Neurosci. Biobehav. Rev.* 36 1934–1951. 10.1016/j.neubiorev.2012.05.010 22669078

[B31] Plichta M. M. Scheres A. (2014). Ventral-striatal responsiveness during reward anticipation in ADHD and its relation to trait impulsivity in the healthy population: a meta-analytic review of the fMRI literature. *Neurosci. Biobehav. Rev.* 38 125–134. 10.1016/j.neubiorev.2013.07.012 23928090 PMC3989497

[B32] Polanczyk G. de Lima M. S. Horta B. L. Biederman J. Rohde L. A. (2007). The worldwide prevalence of ADHD: a systematic review and metaregression analysis. *Am. J. Psychiatry* 164 942–948. 10.1176/ajp.2007.164.6.942 17541055

[B33] Prehn-Kristensen A. Goeder R. Fischer J. Wilhelm I. Seeck-Hirschner M. Aldenhoff J. (2011). Reduced sleep-associated consolidation of declarative memory in attention-deficit/hyperactivity disorder. *Sleep Med.* 12 672–679. 10.1016/j.sleep.2010.10.010 21697007

[B34] Prehn-Kristensen A. Munz M. Goder R. Wilhelm I. Korr K. Vahl W. (2014). Transcranial oscillatory direct current stimulation during sleep improves declarative memory consolidation in children with attention-deficit/hyperactivity disorder to a level comparable to healthy controls. *Brain Stimul.* 7 793–799. 10.1016/j.brs.2014.07.036 25153776

[B35] Prehn-Kristensen A. Munz M. Molzow I. Wilhelm I. Wiesner C. D. Baving L. (2013). Sleep promotes consolidation of emotional memory in healthy children but not in children with attention-deficit hyperactivity disorder. *PLoS ONE* 8:e65098 10.1371/journal.pone.0065098 PMC3667133 23734235

[B36] Rasch B. Born J. (2013). About sleep’s role in memory. *Physiol. Rev.* 93 681–766. 10.1152/physrev.00032.2012 23589831 PMC3768102

[B37] Rechtschaffen A. Kales A. (1968). *A Manual of Standardized Terminology, Techniques and Scoring System for Sleep Stages in Human Subject*. Washington DC: US Government Printing Office, National Institute of Health Publication.

[B38] Ringli M. Souissi S. Kurth S. Brandeis D. Jenni O. G. Huber R. (2013). Topography of sleep slow wave activity in children with attention-deficit/hyperactivity disorder. *Cortex* 49 340–347. 10.1016/j.cortex.2012.07.007 22974674

[B39] Rosenthal R. (1994). “Parametric measures of effect size,” in *The Handbook of Research Synthesis* eds Cooper H. Hedges L. V. (New York, NY: Sage) 231–244.

[B40] Schlarb A. (2011). “CSHQ-DE. screening-fragebogen zu schlafstörungen im kindesalter,” in *Klinisch-psychiatrische Ratingskalen für das Kindes- und Jugendalter* eds Barkmann C. Schulte-Markwort M. Brähler E. (Göttingen: Hogrefe) 128–132.

[B41] Scholle S. Beyer U. Bernhard M. Eichholz S. Erler T. Graness P. (2011). Normative values of polysomnographic parameters in childhood and adolescence: quantitative sleep parameters. *Sleep Med.* 12 542–549. 10.1016/j.sleep.2010.11.011 21601520

[B42] Schwerdtle B. Roeser K. Kübler A. Schlarb A. A. (2010). Validierung und psychometrische Eigenschaften der deutschen version des sleep self report (SSR-DE). *Somnologie* 14 267–274. 10.1007/s11818-010-0496-3

[B43] Silvetti M. Wiersema J. R. Sonuga-Barke E. Verguts T. (2013). Deficient reinforcement learning in medial frontal cortex as a model of dopamine-related motivational deficits in ADHD. *Neural Netw.* 46 199–209. 10.1016/j.neunet.2013.05.008 23811383

[B44] Smith S. W. (1997). *The Scientist and Engineer’s Guide to Digital Signal Processing*. San Diego, CA: California Technical Pub.

[B45] Speekenbrink M. Channon S. Shanks D. R. (2008). Learning strategies in amnesia. *Neurosci*. *Biobehav. Rev.* 32 292–310. 10.1016/j.neubiorev.2007.07.005 17854893

[B46] Swainson R. Rogers R. D. Sahakian B. J. Summers B. A. Polkey C. E. Robbins T. W. (2000). Probabilistic learning and reversal deficits in patients with Parkinson’s disease or frontal or temporal lobe lesions: possible adverse effects of dopaminergic medication. *Neuropsychologia* 38 596–612. 10.1016/S0028-3932(99)00103-7 10689037

[B47] Thomas R. Sanders S. Doust J. Beller E. Glasziou P. (2015). Prevalence of attention-deficit/hyperactivity disorder: a systematic review and meta-analysis. *Pediatrics* 135 e994–e1001. 10.1542/peds.2014-3482 25733754

[B48] Tomasi D. Volkow N. D. (2014). Functional connectivity of substantia nigra and ventral tegmental area: maturation during adolescence and effects of ADHD. *Cereb. Cortex* 24 935–944. 10.1093/cercor/bhs382 23242198 PMC3948497

[B49] Tripp G. Wickens J. (2012). Reinforcement, dopamine and rodent models in drug development for ADHD. *Neurotherapeutics* 9 622–634. 10.1007/s13311-012-0132-y 22806330 PMC3441939

[B50] Tucker M. A. Tang S. X. Uzoh A. Morgan A. Stickgold R. (2011). To sleep, to strive, or both: how best to optimize memory. *PLoS ONE* 6:e21737 10.1371/journal.pone.0021737 PMC3140493 21799746

[B51] van Dongen E. V. Thielen J. W. Takashima A. Barth M. Fernandez G. (2012). Sleep supports selective retention of associative memories based on relevance for future utilization. *PLoS ONE* 7:e43426 10.1371/journal.pone.0043426 PMC3420871 22916259

[B52] Volkow N. D. Wang G. J. Tomasi D. Kollins S. H. Wigal T. L. Newcorn J. H. (2012). Methylphenidate-elicited dopamine increases in ventral striatum are associated with long-term symptom improvement in adults with attention deficit hyperactivity disorder. *J. Neurosci.* 32 841–849. 10.1523/JNEUROSCI.4461-11.2012 22262882 PMC3350870

[B53] Watzlawik M. (2009). Assessing pubertal status with the Pubertal Development Scale: first steps towards an evaluation of a German translation. *Diagnostica* 55 55–65. 10.1026/0012-1924.55.1.55

[B54] Weiß R. H. (2006). *Grundintelligenztest Skala 2 Revision, CFT 20-R*. Göttingen: Hogrefe.

[B55] Wetterling F. McCarthy H. Tozzi L. Skokauskas N. O’Doherty J. P. Mulligan A. (2015). Impaired reward processing in the human prefrontal cortex distinguishes between persistent and remittent attention deficit hyperactivity disorder. *Hum. Brain Mapp.* 36 4648–4663. 10.1002/hbm.22944 26287509 PMC6869504

[B56] Wiebe S. Carrier J. Frenette S. Gruber R. (2013). Sleep and sleepiness in children with attention deficit / hyperactivity disorder and controls. *J. Sleep Res.* 22 41–49. 10.1111/j.1365-2869.2012.01033.x 22762354

[B57] Wiesner C. D. Pulst J. Krause F. Elsner M. Baving L. Pedersen A. (2015). The effect of selective REM-sleep deprivation on the consolidation and affective evaluation of emotional memories. *Neurobiol. Learn. Mem.* 122 131–141. 10.1016/j.nlm.2015.02.008 25708092

[B58] Wilhelm I. Prehn-Kristensen A. Born J. (2012). Sleep-dependent memory consolidation–what can be learnt from children? *Neurosci. Biobehav. Rev.* 36 1718–1728. 10.1016/j.neubiorev.2012.03.002 22430027

[B59] Willcutt E. G. (2012). The prevalence of DSM-IV attention-deficit/hyperactivity disorder: a meta-analytic review. *Neurotherapeutics* 9 490–499. 10.1007/s13311-012-0135-8 22976615 PMC3441936

[B60] Yaroush R. Sullivan M. J. Ekstrand B. R. (1971). Effect of sleep on memory. II. Differential effect of the first and second half of the night. *J. Exp. Psychol.* 88 361–366. 10.1037/h0030914 4326302

[B61] Yoshimasu K. Barbaresi W. J. Colligan R. C. Voigt R. G. Killian J. M. Weaver A. L. (2012). Childhood ADHD is strongly associated with a broad range of psychiatric disorders during adolescence: a population-based birth cohort study. *J. Child Psychol. Psychiatry* 53 1036–1043. 10.1111/j.1469-7610.2012.02567.x 22647074 PMC3608464

[B62] Zimmermann P. Gondan M. Fimm B. (2002). *KiTAP, Kinderversion der Testbatterie zur Aufmerksamkeitsprüfung*. Herzogenrath: Psytest.

